# Multi-modal imaging of the subscapularis muscle

**DOI:** 10.1007/s13244-016-0526-1

**Published:** 2016-10-17

**Authors:** Mona Alilet, Julien Behr, Jean-Philippe Nueffer, Benoit Barbier-Brion, Sébastien Aubry

**Affiliations:** 1 Department of Musculoskeletal Imaging, CHRU de Besançon, CHRU Jean Minjoz, Boulevard Fleming, 25030 Besançon Cedex, France; 2 Anatomy Laboratory, University of Franche-Comte, Besançon, France; 3 Groupe CIMRAD, Centre d’imagerie des Tilleroyes, Besançon, France; 4 Nanomedicine and Imagery Laboratory, EA4662, University of Franche-Comte, Besançon, France

**Keywords:** Subscapularis, Tendon injury, Rotator cuff, Magnetic resonance imaging, Coracoid impingement

## Abstract

**Abstract:**

The subscapularis (SSC) muscle is the most powerful of the rotator cuff muscles, and plays an important role in shoulder motion and stabilization. SSC tendon tear is quite uncommon, compared to the supraspinatus (SSP) tendon, and, most of the time, part of a large rupture of the rotator cuff. Various complementary imaging techniques can be used to obtain an accurate diagnosis of SSC tendon lesions, as well as their extension and muscular impact. Pre-operative diagnosis by imaging is a key issue, since a lesion of the SSC tendon impacts on treatment, surgical approach, and post-operative functional prognosis of rotator cuff injuries. Radiologists should be aware of the SSC anatomy, variability in radiological presentation of muscle or tendon injury, and particular mechanisms that may lead to a SSC injury, such as coracoid impingement.

***Teaching Points*:**

• *Isolated subscapularis (SSC) tendon tears are uncommon.*

• *Classically, partial thickness SSC tendon tears start superomedially and progress inferolaterally.*

• *Long head of biceps tendon medial dislocation can indirectly signify SSC tendon tears.*

• *SSC tendon injury is associated with anterior shoulder instability.*

• *Dynamic ultrasound study of the SSC helps to diagnose coracoid impingement.*

## Introduction

The subscapularis (SSC) muscle is one of the four components of the rotator cuff along with the supraspinatus (SSP), infraspinatus, and teres minor muscles. The long head of biceps (LHB) tendon is classically associated.

The muscles of the rotator cuff and their tendinous insertions play an important role in shoulder stability, and in the motion of the glenohumeral joint. The SSC in particular has the greatest force-producing capacity, followed by the infraspinatus, SSP, and teres minor [1].

Rotator cuff pathologies are very common, and foremost among these are SSP tendon tears. SSC tendon pathology is quite uncommon, and most of the time, occurs as part of a larger rupture of the rotator cuff.

In cadaveric studies, the prevalence of SSC tendon tears varies between 29 and 37 % [2], whereas it is estimated to be between 5 and 27 % in clinical studies [3, 4]. This discrepancy could be related to the incomplete visualization of the SSC tendon on both arthroscopy and open surgery [5].

Pre-operative diagnosis of SSC tendon tears is therefore a major issue, especially since a lesion of the SSC tendon will have an impact on treatment, surgical approach, and post-operative functional prognosis [6]. The purposes of this article are to describe the normal appearance of the SSC, and to review the imaging findings encountered in patients with lesions of the SSC tendon and muscle, according to specific pathophysiological mechanisms.

## Anatomy

**Fig. 1:**
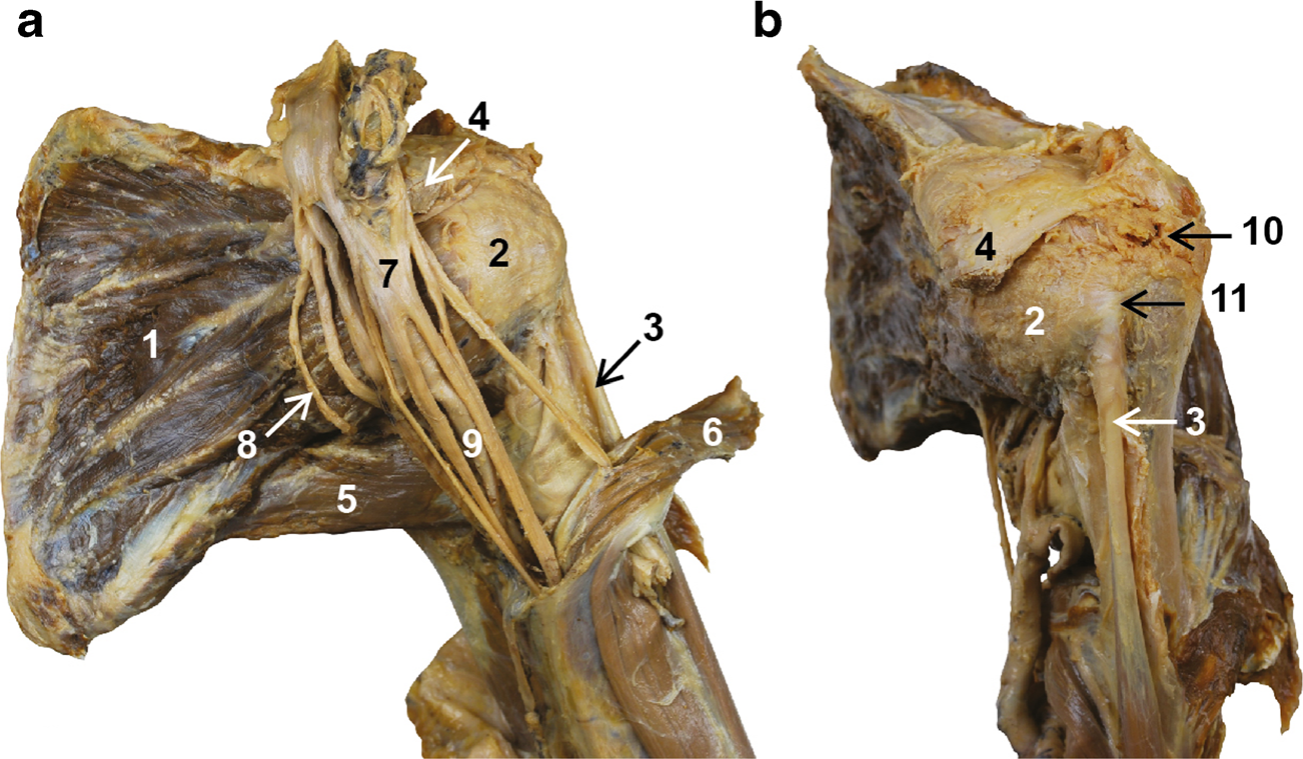
Anatomical views of the shoulder: front view (**a**), lateral oblique view (**b**). 1: Subscapularis muscle. 2: Subscapularis tendon. 3: long head of biceps tendon. 4: Coracoid process. 5: Teres major muscle. 6: Coraco-brachialis muscle. 7: Brachial plexus. 8: Lower subscapularis nerve. 9: Axillary artery

The SSC muscle is the largest and the most powerful of the rotator cuff muscles. It is located in the subscapular fossa at the anterior aspect of the scapula. It contains multiple tendinous and muscular bundles that merge laterally into a flattened tendon in the upper two-thirds of the muscle. This tendinous portion has a variable insertion, mostly on the lesser tuberosity of the humerus, but also on the greater tuberosity and the bicipital groove [2]. SSC tendon fibres contained within a fibrous expansion from the pectoralis major tendon form the transverse ligament, which is a fascia covering the vertical portion of the LHBT (Fig. 1).

The lower third of the muscle has a muscular attachment onto the inferior part of the lesser tuberosity and onto the anterior aspect of the humeral diaphysis.

### Innervation

Variants of the innervation of the SSC muscle are common [7]. Classically, the two superior thirds and the inferior third of the SSC muscle are innervated, respectively, by the upper and lower subscapularis nerves, which are both branches of the posterior chord of the brachial plexus (Fig. 1). Electromyographic (EMG) studies have shown differences in activity between the upper and lower portion of the SSC muscle, suggesting that they work as two different muscular units during voluntary shoulder movements [8].

### Function

The principal role of the SSC muscle in shoulder motion is internal rotation. In variable positions, the superior subscapularis fibres assist in abduction, while the inferior fibres assist in adduction. At the beginning of elevation shoulder movement, EMG onset of the upper portion of the SSC occurs first, which increases the glenohumeral congruence. The upper SSC also demonstrates higher levels of activation than the lower portion, which confirms the hypothesis of a participation in abduction [8].

The SSC muscle provides active stabilization of the shoulder during external rotation and flexion [9]. Its action is coordinated with the other muscles of the rotator cuff to ensure shoulder function and the centring of the humeral head in the glenoid fossa. There are balanced forces between the superior fibres of the SSC and its antagonist, the infraspinatus muscle, in the axial plane. Conversely, the inferior fibres of the SSC resist, in the coronal plane, the elevation of the humeral head induced by the deltoid muscle [2].

The SSC muscle also plays a role in passive stability of the glenohumeral joint, resisting anterior luxation. Indeed, anterior luxation is associated with laxity of the lower portion of the SSC muscle [10]. This explains why some authors recommend surgical repair of the SSC for treatment of anterior glenohumeral instability [11]. Also, the SSC tendon stabilizes the LHBT within the rotator interval *(see below).*

### Anatomical relations

#### Rotator interval

**Fig. 2:**
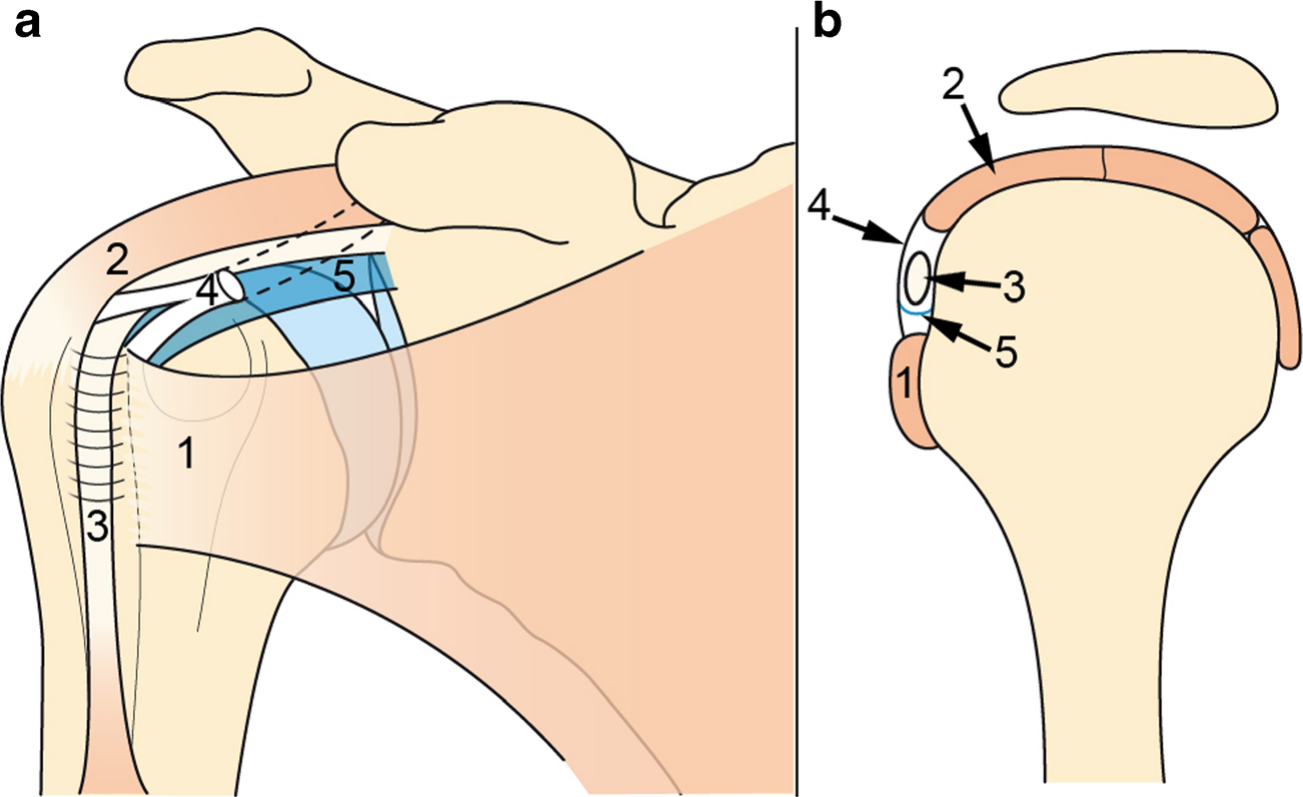
Schematic representation of the rotator interval (RI): front view (**a**), sagittal view (**b**). The RI is bordered by the SSC tendon inferiorly (1) and the SSP tendon superiorly (2) and contains the intra-articular portion of LHBT (3). CHL (4) and SGHL (5) surround the LHBT in the RI, acting as a pulley system for LHBT stabilization

The SSC tendon contributes to the formation of an anatomical space located in the anterosuperior portion of the shoulder, called the rotator interval (Fig. 2), which is a tendinous gap in the rotator cuff, exclusively covered by capsular tissue. It is a triangular shaped space bordered inferiorly by the superior free edge of the SSC tendon, and superiorly by the anterior free edge of the SSP tendon. The base of this triangle is formed by the coracoid process medially, and its apex is the intertubercular sulcus laterally [12]. The anterior aspect of the rotator interval is formed by a fibrous capsule made of blended fibres coming from the SSC and SSP tendons. It is reinforced by two ligaments, namely the coracohumeral ligament (CHL) and the superior glenohumeral ligament (SGHL). The inferomedial CHL, the SGHL and the superior fibres of the SSC tendon unite in the lateral rotator interval and act as a pulley system for the LHBT, which is a key element for stabilization of the LHBT [13]. Indeed, this pulley system prevents inferior luxation of the horizontal portion of the LHBT, and medial luxation of its vertical portion. The most superior insertion point of the SSC could be the most important structure for medial stabilization of LHBT [14].

#### Other anatomical relations

The origin of the vascular-nervous pedicle of the upper limb is located at the anterior aspect of the SSC muscle. The axillary artery and vein, and the chords of the brachial plexus lean on the SSC muscle. In front of the SSC muscle, the medial, lateral and posterior chords of the brachial plexus divide into their terminal branches (Fig. 1).

Among the anatomical variations of the shoulder, an accessory coracobrachialis muscle that crosses in front of the SSC tendon is observed in 1 % of cases [15].

Also, in the coronal plane there is a continuity between the SSS muscle, inserted in the subscapularis fossa, and the teres major, inserted on the lateral aspect of the scapula (Fig. 1).

## Clinical features

Most rotator cuff tears are asymptomatic [16]. When a SSC tendon lesion is symptomatic, spontaneous or triggered pain is preferentially located in the anterior region of the shoulder. A tear is painful during development, whereas a constituted tear is less painful, if at all. When subacromial-subdeltoid (SASD) bursitis is associated, pain is common. Calcific tendinitis can also be painful, especially when hydroxyapatite calcifications are in the resorptive phase [17].

Most of the time, clinical examination can orientate towards an articular or periarticular pathology, but it cannot identify the precise topography or the pathological mechanism, even if there are many clinical tests [18]. The SSC tendon is evaluated by Gerber’s “lift-off test” [19], or the “Belly press test”. It can also be tested by evaluating resisted internal rotation at maximal abduction (90°) or maximal external rotation [20]. Finally, when the SSC tendon is torn, external rotation of the shoulder will be increased in passive mobilization [19].

## Pathogenesis of SSC tendon lesions

Several factors can concur to weaken the SSC tendon. Rarely, in young patients, SSC tears can occur on a healthy tendon and result from kinetic trauma in traction. Frequently, SSC tendon tears occur on underlying chronic tendinopathy, which refers to tendinosis on histopathology. When these tendinosis phenomena are associated with calcium deposits within the substance of the tendon, this entity is called calcific tendinitis. Furthermore, extrinsic factors can generate excessive mechanical constraints on the SSC tendon. Among these, coracoid or anteromedial impingement and anterior instability of the shoulder are secondary to specific pathophysiological mechanisms that lead to the findings detailed below.

## Radioanatomy

### Plain radiographs

To explore the shoulder, anteroposterior views with three projections (neutral, external and internal rotation), an axillary view, and a scapular “Y” view must be performed. The axillary view can show alterations of the lesser tuberosity, such as irregularities or cysts, which argue in favour of a SSC tendon lesion [21]. Anterior subluxation of the humeral head can also be seen on this view [22]. The “Y” view is helpful to locate tendinous calcifications and to look for a narrow subcoracoid space, which can both lead to coracoid impingement (see below).

### Ultrasound

**Fig. 3:**
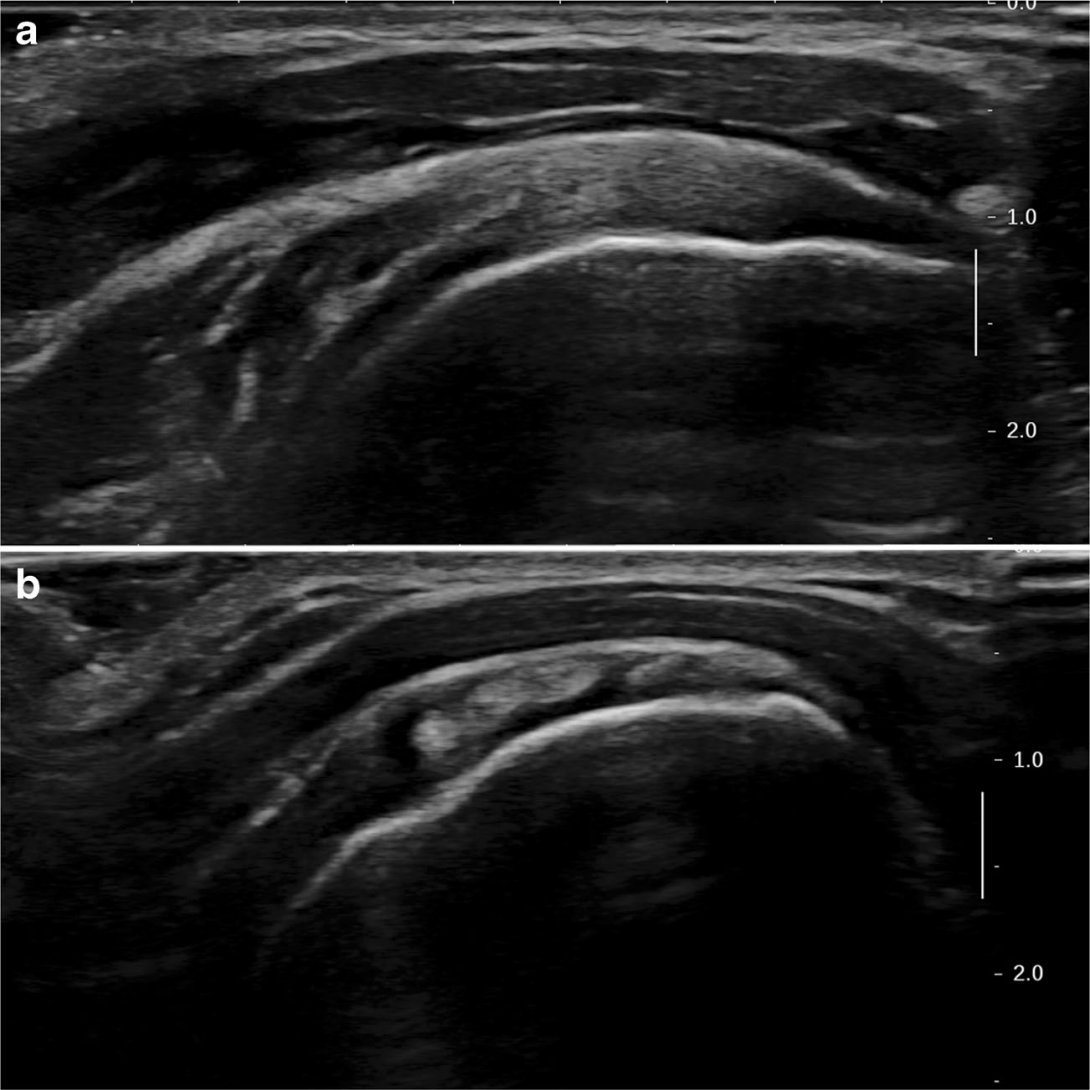
B-mode ultrasound images of the SSC. **a** Transverse view (long axis) : the normal SSC tendon is hyperechoic compared to the adjacent muscle and has a fibrillary structure. **b** Sagittal view (short axis): multi-pennate structure of the SSC tendon with alternation of hypo and hyperechoic zones

The SSC tendon is first evaluated in its long axis, by placing the probe transversally over the anterior shoulder, in the same position as for the study of the LHBT. External rotation of the forearm, while keeping the elbow next to the chest, brings out the lesser tuberosity and SSC tendon laterally. The normal SSC tendon moves freely under the coracoid process and the LHBT must stay in its groove during external rotation. Next, a short axis view of the SSC tendon is obtained by turning the probe 90°, which demonstrates its multi-pennate structure (Fig. 3).

### MRI

MRI images must be obtained with a dedicated shoulder coil. The shoulder is studied in three planes (i.e., axial, oblique coronal, oblique sagittal) with fat-suppressed (FS) fluid-sensitive sequences (proton density (PD) or T2 weighted). Axial and oblique sagittal planes are the most informative for the SSC tendon. The axial plane evaluates the SSC tendon in its long axis and makes it possible to explore the LHBT and its position within the intertubercular sulcus. The short axis of the SSC tendon and the multi-pennate structure of the SSC muscle are studied in the oblique sagittal plane.

An oblique sagittal T1-weighted sequence is also recommended to evaluate fatty infiltration and muscle mass.

### Magnetic resonance arthrography (MRA)

An accurate and a traumatic shoulder arthrography technique is essential to avoid false images of SSC tendon lesions. The anterior approach targeting the rotator cuff interval under ultrasound or fluoroscopic guidance is currently the most widely performed technique [23], even if a posterior approach makes it possible to avoid any iatrogenic contamination of the SSC tendon or the SASD bursa. Ten to twelve cc of contrast medium (dilution of gadolinium at 0.0025 mmol/L) are injected into the glenohumeral joint. The MRA acquisition protocol includes FS T1-weighted in three planes (or a 3D-FS-T1_w_), a coronal FS T2-weighted or PD-weighted sequence, and a T1-weighted sagittal sequence. In healthy patients, the contrast agent surrounds the articular surface of the SSC tendon, and underlines the LHBT in its groove, without any leak of contrast agent in front of the lesser tuberosity, or in the SASD bursa.

### Computed tomography arthrography (CTA)

The same technique of arthrography outlined above is also performed for CTA. Ten to twelve cc of contrast medium are injected into the glenohumeral joint (nonionic iodine agent at 200–300 mgI/mL). Spiral scanning using a small field of view allows isotropic data acquisition and mutiplanar reformation. CTA allows a precise analysis of SSC tendon tears thanks to its high resolution and high positive contrast between the tendon and contrast medium.

## Pathological features

### Tendinopathy

**Fig. 4:**
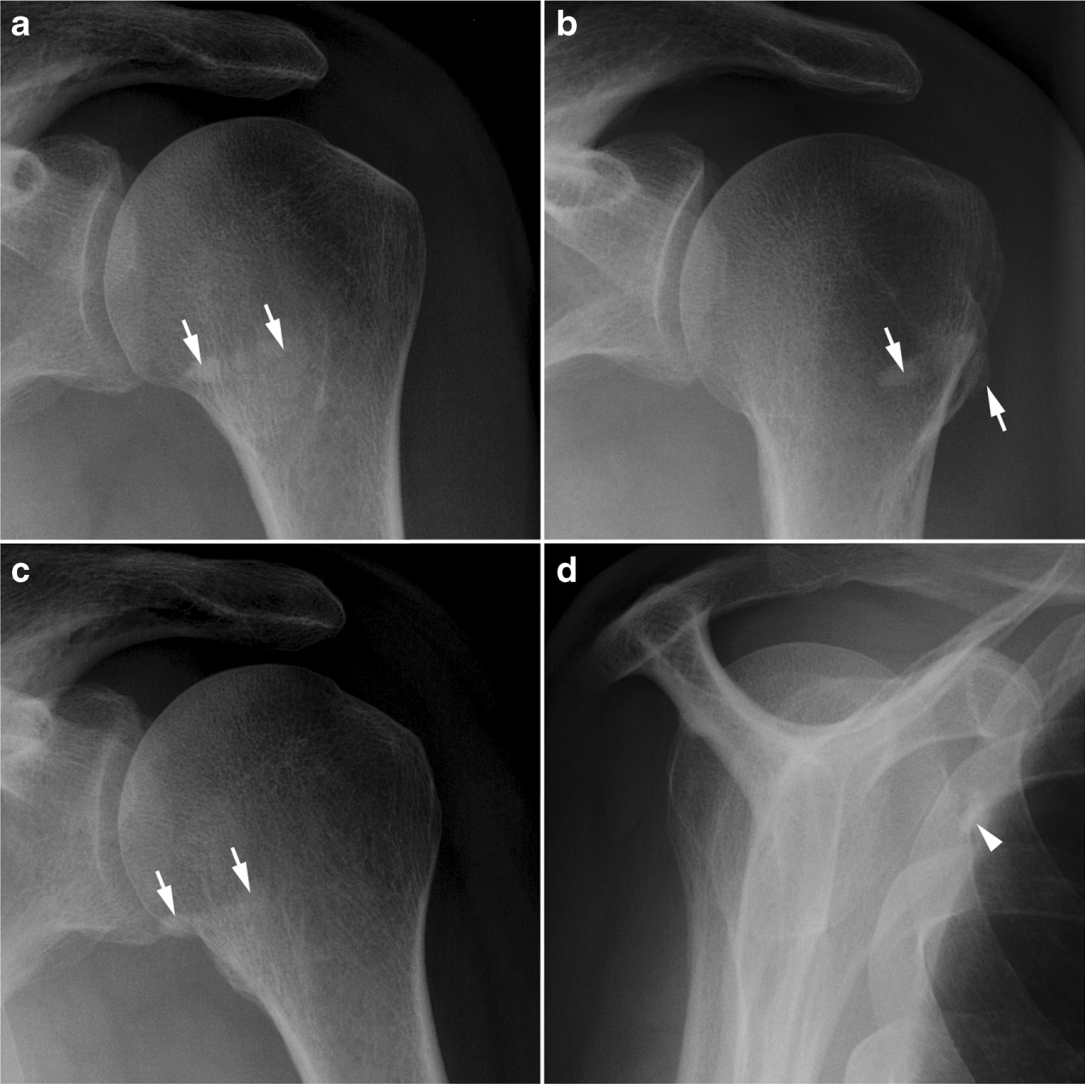
Calcific tendinitis of the SSC. Calcificationsof the SSC tendon (white arrows) overlap the lesser tuberosity in the front view in neutral rotation (**a**), are lateralized in external rotation (**b**), and medialized in internal rotation (**c**). On the Y view, calcifications of SSC tendon are seen under the coracoid process (white arrowhead) (**d**)

In calcific tendinitis, plain radiographs allow localization and assessment of the texture and morphology of calcific deposits (Fig. 4). Ultrasound examination is also a fundamental tool in the diagnosis of calcific tendinitis. It shows a hypoechoic SSC tendon that loses its fibrillary echo structure. It can also show associated calcification, and specify its topography and morphology. Voluminous calcifications can be responsible for coracoid impingement, which must be investigated using dynamic study in internal rotation. Increased signal in colour Doppler is correlated with intensity of symptoms [24].

MRI provides no additional information for the evaluation of calcific tendinitis. It shows a non-fluid intermediary high signal on T2 or PD_w_ sequences [25]. Calcifications appear as low signal in all sequences and can therefore be missed. High signal on T2_w_images around calcifications can be seen because of oedema at the resorptive phase [26].

### SSC tendon tears

**Fig. 5:**
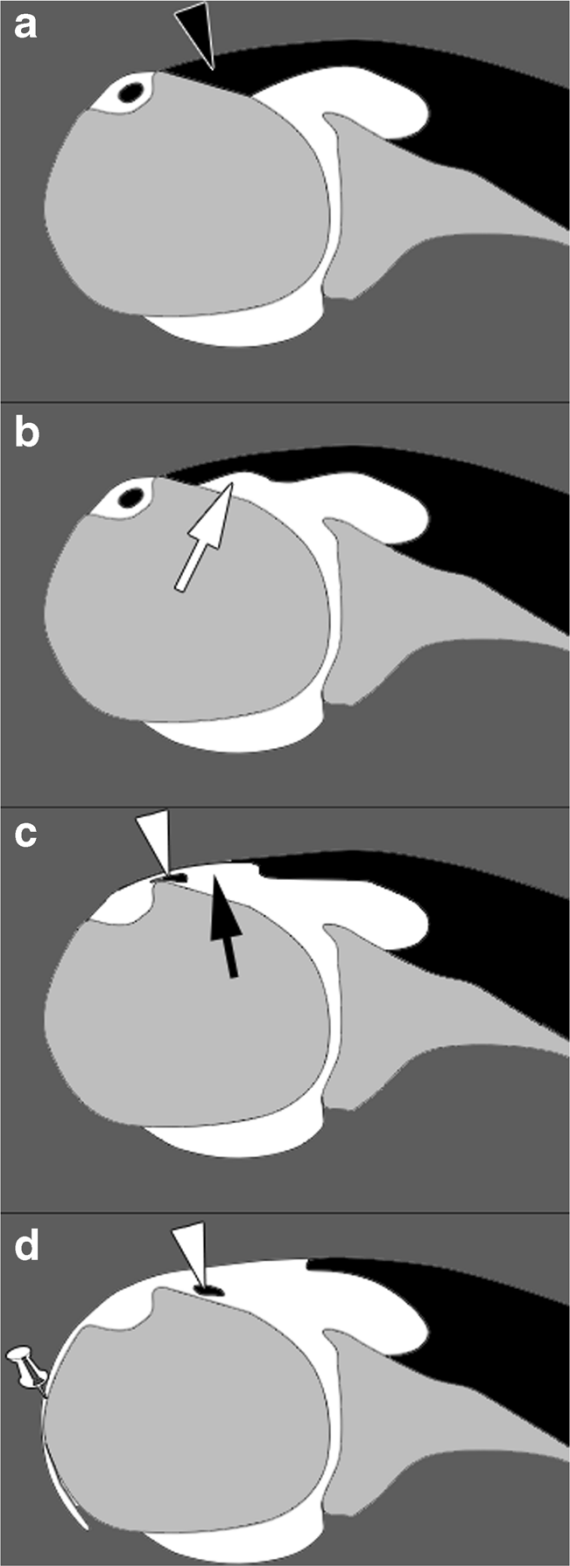
Schematic representation of SSC tendon tears on magnetic resonance or computed tomography arthography. Normal SSC tendon (black arrowhead) (**a**). Incomplete partial thickness tear (white arrow) (**b**). Complete partial thickness tear (black arrow) associated with LHBT (white arrowhead) subluxation (**c**). Complete full thickness tear associated with LHBT (white arrowhead) dislocation and opacification of the SASD bursa (pin) (**d**)

**Fig. 6:**
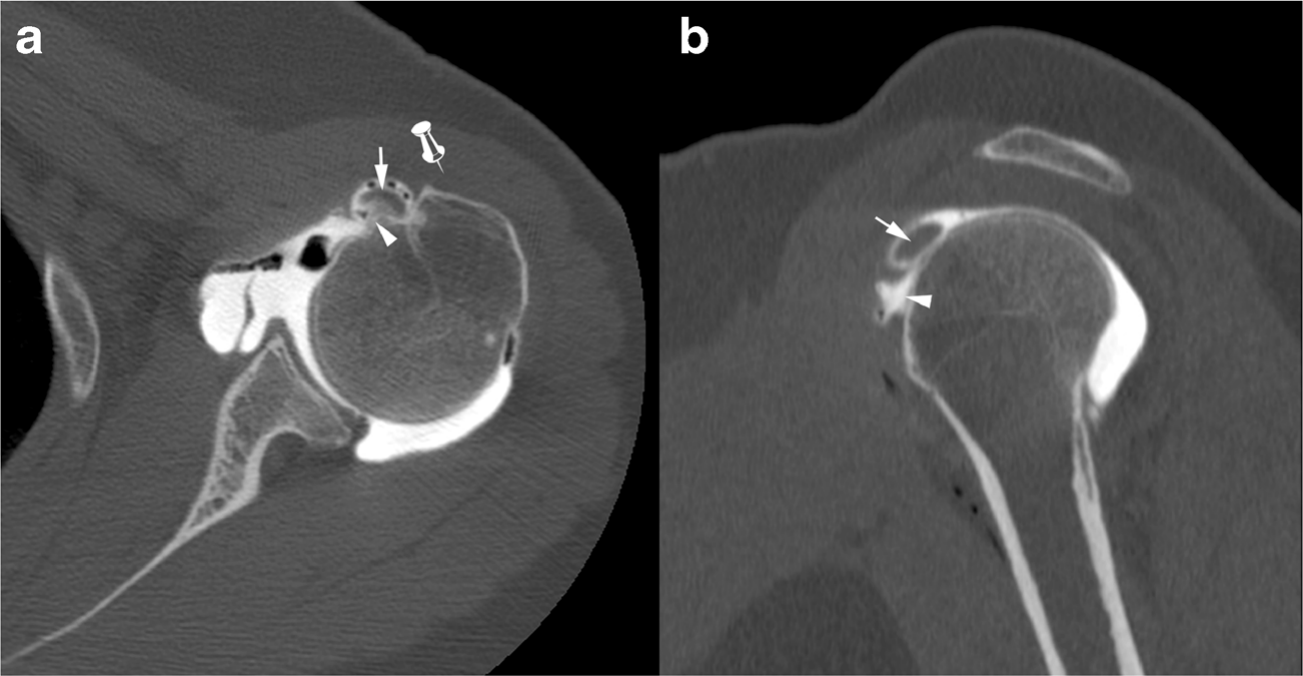
Computed tomography arthrography of the left shoulder with axial (**a**) and sagittal oblique (**b**) multiplanar reconstructions in a 38-year-old man, in a post-traumatic context. Isolated complete partial thickness tear of the SSC tendon, located at the articular side of the tendon, involving the upper fibres (arrow head), responsible for subluxation of the LHBT (arrow). Absence of contrast in the SASD bursa (pin) confirms the absence of transfixing or full thickness tear

Most SSC lesions (Fig. 5) are part of an extended rotator cuff tear. They can also occur in anterosuperior lesions of the rotator cuff, involving the rotator interval, and the SSP, SSC, and LHB tendons. There are 80 % of SSC tendon tears associated with a SSP tear [27]. Isolated SSC tendon tears are uncommon (Fig. 6) and occur in specific mechanisms (traumatism, glenohumeral instability) [28].

These tears are best categorized as full-thickness if communicating with the SASD (partial-thickness if not) and complete if the whole tendon is disrupted (incomplete if not). Most of the time, SSC tendon tears are partial thickness tears involving the articular side of the tendon. On ultrasound, partial thickness tears appear as a hypoechoic tendon, with focal interruption of deep fibres, at the articular side of the tendon [29]. MRI shows a local high signal on FS T2-weighted images, without any extension to the bursal surface. The most sensitive sign in CTA and MRA is the presence of contrast agent in front of the lesser tuberosity [28]. Absence of opacification of the SASD bursa rules out a full thickness tear.

**Fig. 7:**
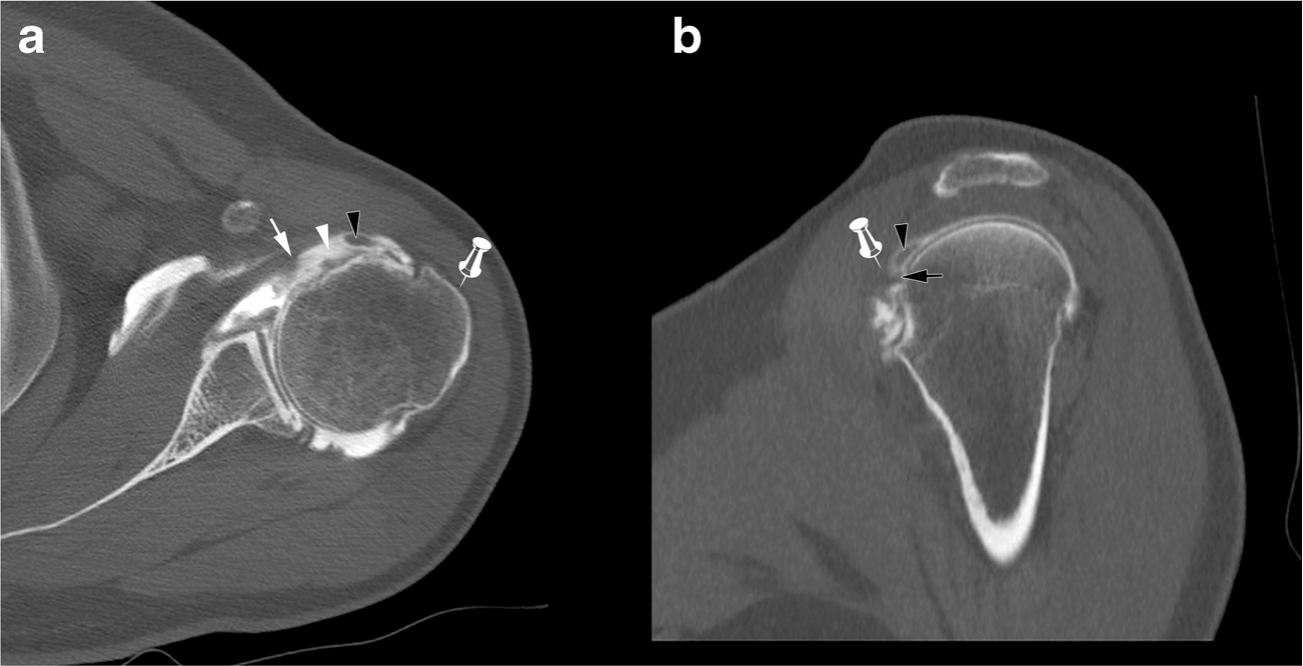
Forty-five-year old man with pain of the anterior shoulder. Computed tomography arthrography of the left shoulder with axial (**a**) and sagittal oblique (**b**) reconstruction. Complete partial thickness tear of the SSC tendon as shown by the absence of opacification of SASD bursa (pin), and the whole disruption of the tendon in the lateral direction (arrow head). The tendinous stump presents an intermediary retraction (arrow). This tear leads to a medial subluxation of the LHBT (black arrowhead) (**a**). Note that an intact superior glenohumeral ligament (black arrow) prevents the LHBT from inferior dislocation (**b**)

Partial thickness tears start at the superomedial aspect of its enthesis and progress inferolaterally [22]. As a consequence, incomplete partial thickness tear (where only the superomedial fibers are disrupted) evolve into complete partial thickness tear (where the whole tendon is disrupted but its superficial aponeurosis and transverse ligament) (Fig. 6). In the latter case, MRA and CTA show a continuity between the intra-articular contrast and the contrast in the bicipital groove (Fig. 7). The SSC tendon can stay in its place, maintained by the transverse ligament, or can be retracted.

Full thickness tears, whether partial or complete, are less common. Since the transverse ligament is also injured in these cases, there is a communication between the glenohumeral joint and the SASD bursa. In partial full thickness tears, only some fibres of the SSC tendon are injured, whereas complete full thickness tears involve all the SSC tendon fibres. This kind of tear is more frequent when there is a history of trauma, and beyond the age of 40 years [30].

Ultrasound shows a hypoechoic or anechoic defect from the articular to bursal surface of the SSC tendon. MRI shows a fluid high signal on T2_w_images involving the full thickness of the SSC tendon. Opacification of the SASD bursa is observed on MRA or CTA because of the transfixing tear.

**Fig. 8:**
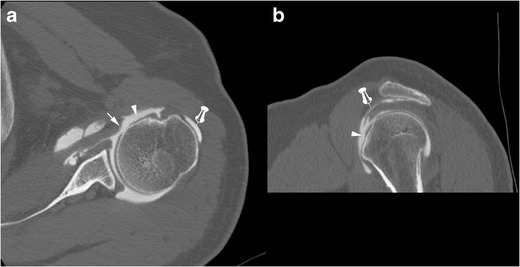
Computed tomography athrography of the left shoulder with axial (**a**) and sagittal oblique (**b**) multiplanar reconstructions. Opacification of the SASD (pin) and whole disruption, either laterally (**a**) or vertically (**b**) (arrow heads) of the SSC tendon are consistent with a complete full thickness tear. Intermediate retraction of the tendinous stump (arrow)

**Fig. 9:**
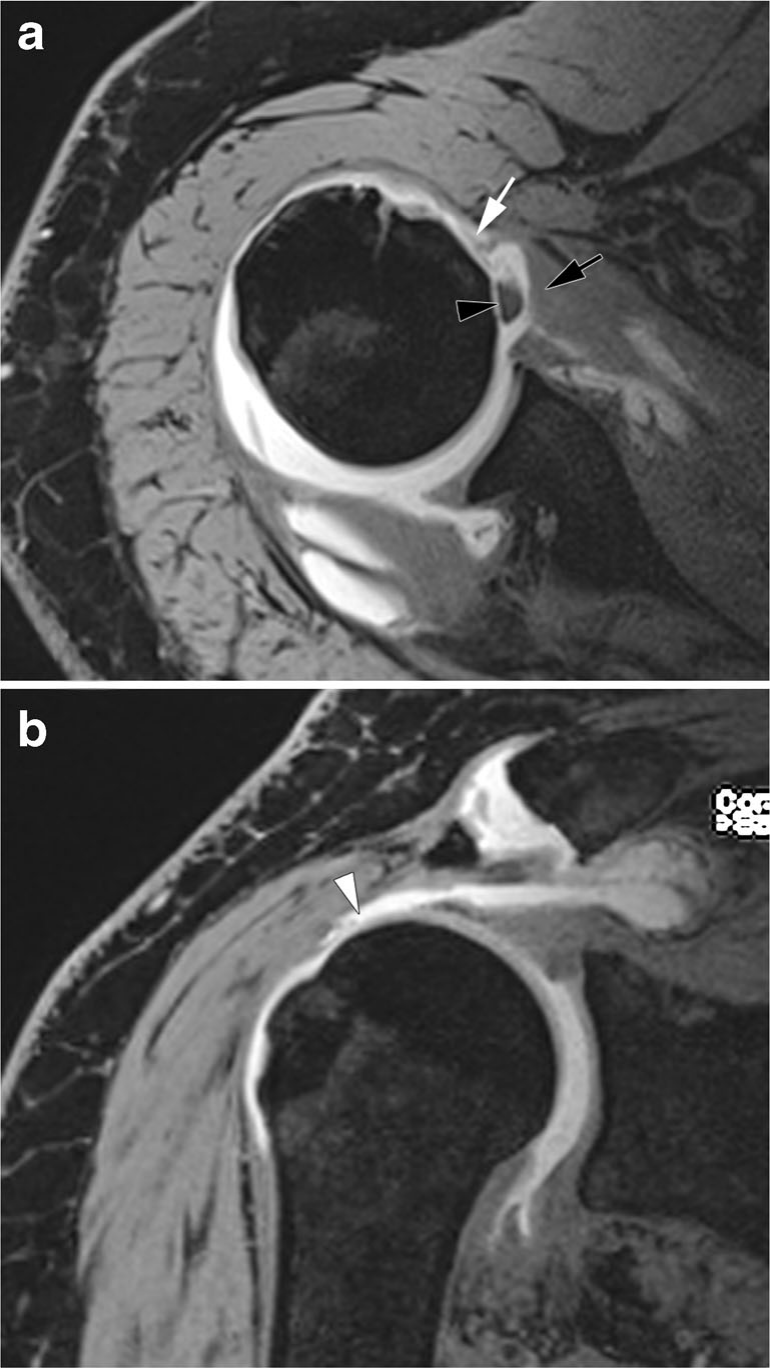
Axial (**a**) and sagittal oblique reformats (**b**) from a 3D FS T1-weighted MRA. Massive rotator cuff tear as shown by complete full thickness tears of the SSC (white arrow) (**a**) and the SSP (white arrow head) tendons (**b**). LHBT is medially dislocated (black arrow head) (**a**), and there is a proximal retraction of SSC tendon (black arrow)

In case of a complete full thickness SSC tendon tear, tendinous stump retraction must be evaluated: it can be located in front of the anatomical neck (intermediate retraction) (Fig. 8) or the glenohumeral joint (proximal retraction) (Fig. 9) [22].

**Fig. 10:**
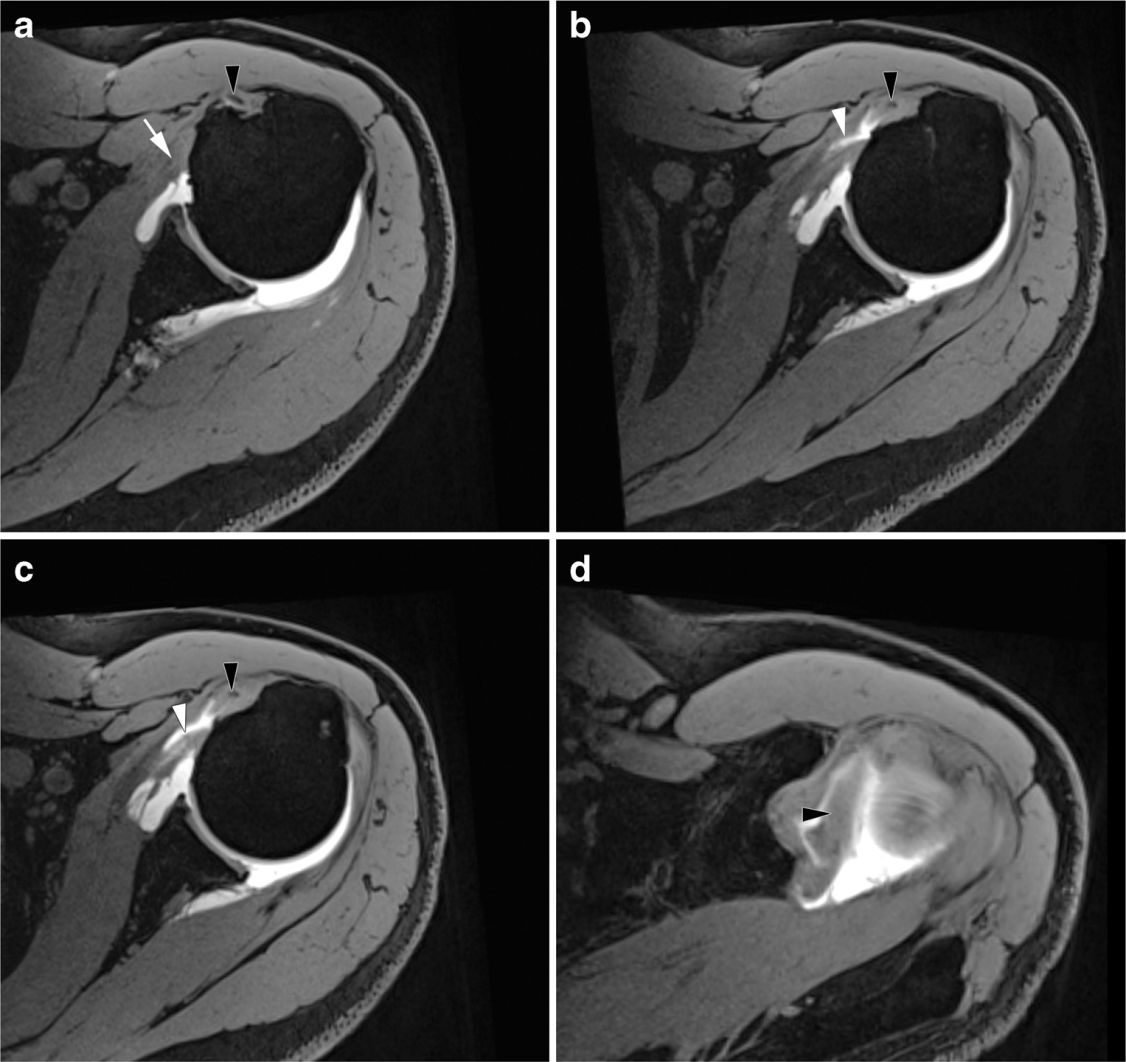
Axial FS T1 weighted MRA images (**a**, **b**, **c**) and axial oblique reformat through the LHBT (**d**). The inferior portion of the SSC tendon is normal (white arrow) (**a**), but there is an incomplete partial tear of the bursal side of the superior portion of the SSC tendon. This is responsible for a medial subluxation of the LHBT (black arrow head). Superiorly, the horizontal portion of the LHBT is hypertrophic (black arrow head), consistent with an “hourglass” biceps

**Fig. 11:**
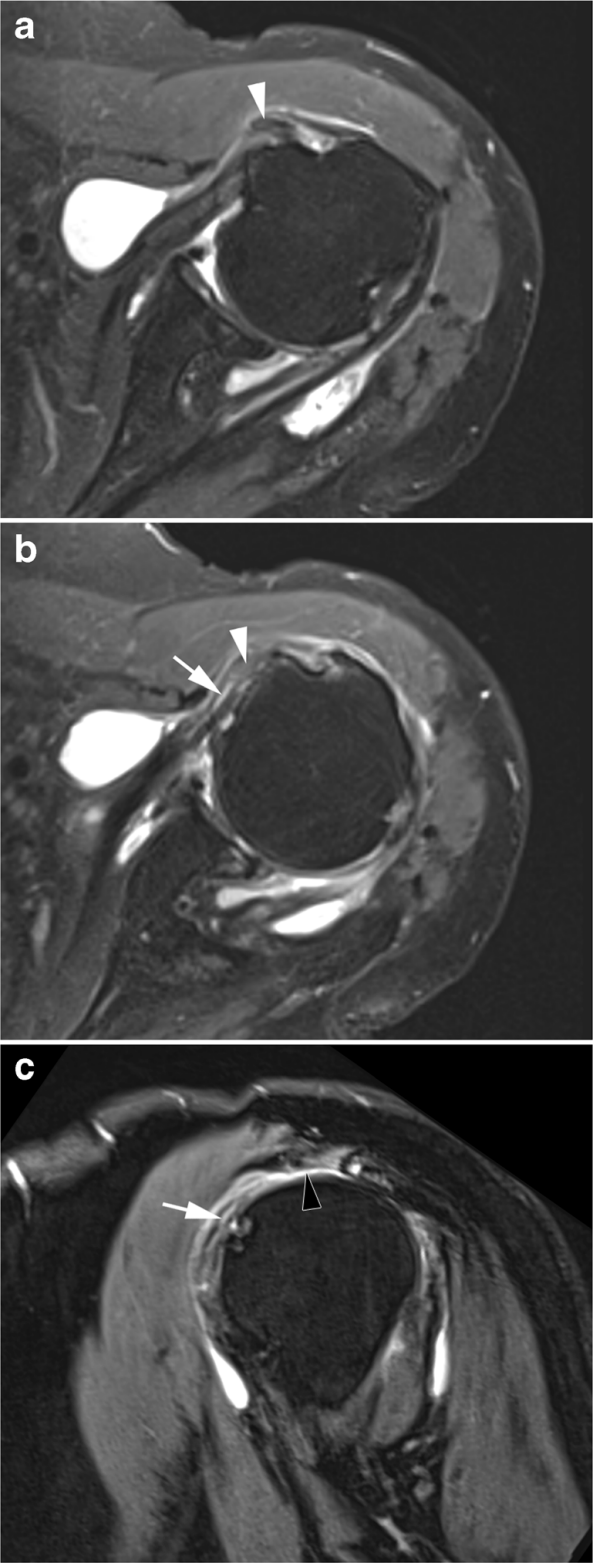
Left shoulder MRI of a 76-year-old woman. On axial FS PD-weighted images, the LHBT (white arrow heads) is medially displaced (**a**), and incarcerated into a cleavage of the SSC tendon (white arrow (**b**). On the sagittal oblique FS T2-weighted image (**c**), the SSC tendon tear (white arrow) is associated with a complete full thickness tear of the SSP tendon (black arrow head)

More rarely, there may be partial thickness tears at the bursal side of the tendon (Fig. 10), and/or horizontal cleavage tear of the SSC tendon (lamellar dissection), with the LHBT inserting into the cleavage [22]. It is an example of a hidden lesion, referring to an injury of the biceps pulley whose arthroscopic and clinical diagnosis is challenging (Fig. 11).

The sensitivity and specificity of ultrasound, MRI, CTA, and MRA for SSC lesions are detailed in Table 1.

**Table 1:** Sensitivity and specificity of imaging techniques for the diagnosis of SSC tendon tears

Imaging technique	Sensitivity	Specificity	Reference
Ultrasound	39.5 %	93.1 %	[31]
MRI	83 %	70 %	[32]
MR-arthrography	81–83 %	82–83 %	[33]
CT-arthrography	64.7 %	98.2 %	[34]

*MRA* magnetic resonance arthrography, *CT* computed tomography

**Fig. 12:**
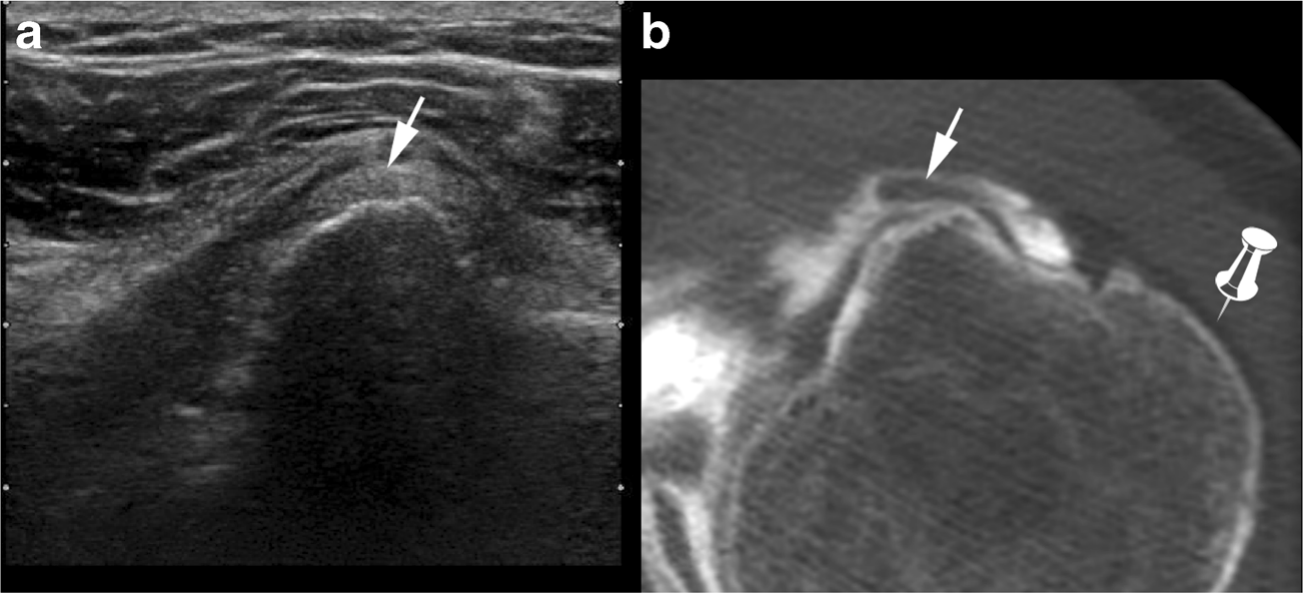
Ultrasound (**a**) and computed tomography (CT) arthography (**b**) correlation: complete partial thickness tear of the upper SSC tendon responsible for subluxation of the LHBT (white arrow), located in front of the medial edge of the intertubercular sulcus. On CT-arthrography (**b**), contrast in the intertubercular groove is in direct continuity with intra-articular contrast, without any opacification of the SASD bursa (pin) because the bursal side of the transverse ligament is intact

Injury of the LHBT is associated in 50 % cases of SSC tendon tears [22]. The LHBT can be dislocated (in front of the lesser tuberosity and sometimes into the glenohumeral joint), or sub-dislocated (LHBT located in front of the internal aspect of the bicipital groove) (Fig. 12). The LHBT can also be torn. Indirect ultrasound features of LHBT sub-dislocation are widening and flattening of the LHBT [22]. Moreover, the anechogenic triangle separating the LHBT from the internal aspect of the bicipital groove is no longer visualized (Brasseur’s triangle sign).

**Fig. 13:**
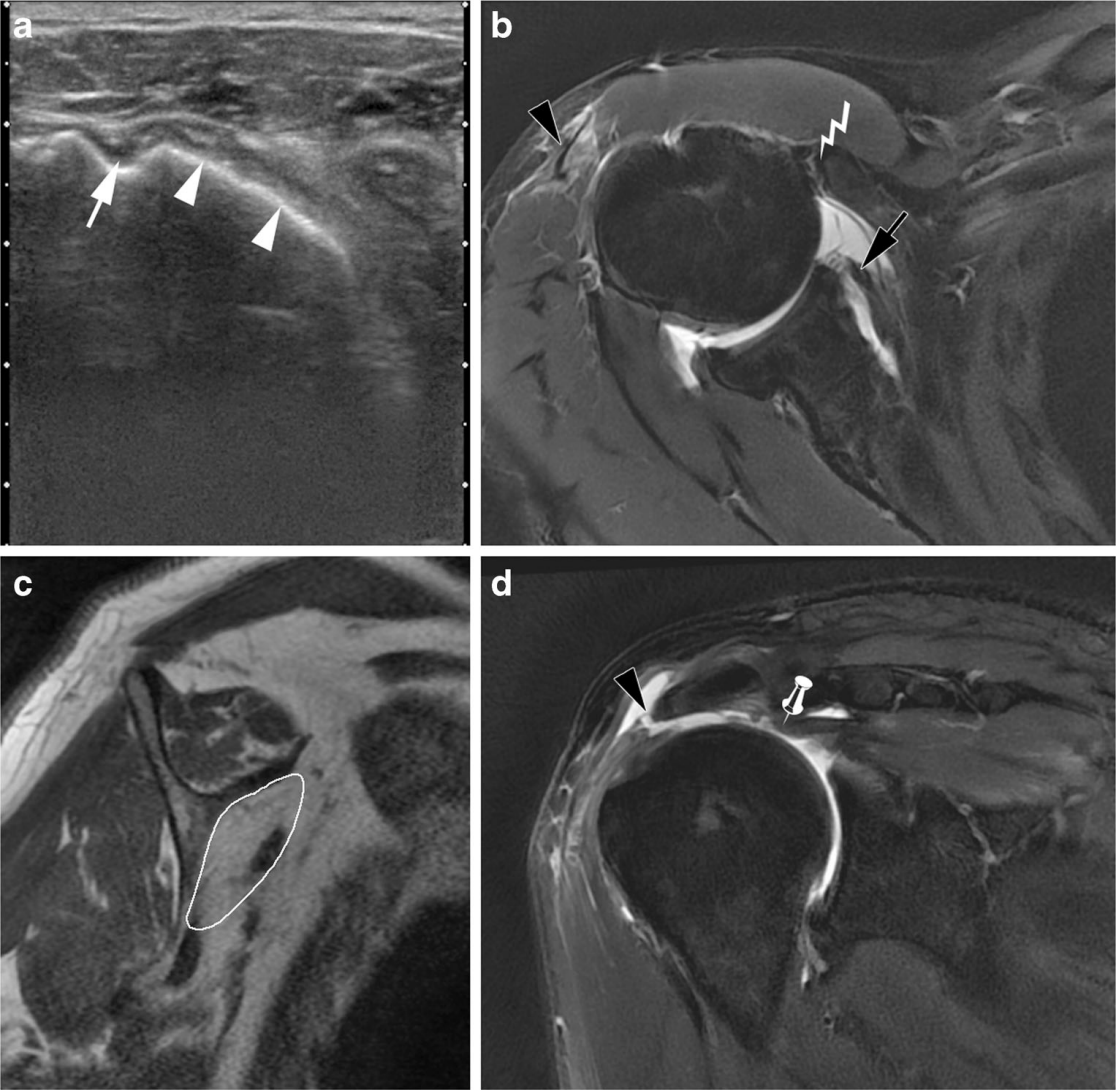
Sixty-two-year old man with a massive right rotator cuff tear involving the SSP, LHBT and SSC tendons. Transverse US image (**a**): exposure of the lesser tuberosity (white arrow heads) consistent with a complete full thickness tear of the SSC tendon. The intertubercular sulcus (white arrow) is empty because the LHBT is torn. Axial FS DP weighted MRI image (**b**): proximal retraction of the torn SSC tendon (black arrow). On sagittal T1 weighted MRI images (**c**) there is severe amyotrophy and fatty degeneration of the SSC muscle (white outline). On coronal FS DP weighted MRI images, there is an intermediate retraction of the torn SSP tendon (pin) (**d**). As the SSC tendon can no longer fulfil its stabilizing role, there is passive instability and an anterior sub-dislocation of the humeral head coming in contact with the coracoid process (lightning) (**b**). Also note an associated tear of the acromial head of the deltoid tendon (black arrow heads) (**b**, **d**)

Fatty degeneration of the SSC muscle is usually evaluated on CT or MRI (Fig. 13) using Goutallier’s grading system [35]. Quantitative measurement of Hounsfield units within the SSC muscle on sagittal CT images correlates well with Goutallier grades and may also be used [36]. Conversely, measurement of signal intensity in MRI is not reliable for the evaluation of fatty degeneration [37]. Experience shows that fatty degeneration is often greater in the upper part of the SSC muscle, probably because the SSC tendon tears begin superiorly.

There is a significant correlation between Goutallier grades and rupture rate after surgical repair (i.e., rerupture) : while it is <10 % in grade 0 and 1, it increases to 28 % in grade 2, and 50 % in grade 3 [4]. Rerupture rate is also correlated with the delay between injury and surgery [4]. In post-traumatic SSC tendon tears, early arthroscopic repair (within an average of 3.7 months) leads to good functional results, a decrease in fatty degeneration and improvement of muscle mass on imaging [38].

### SSC tendon lesions and anterior instability

**Fig. 14:**
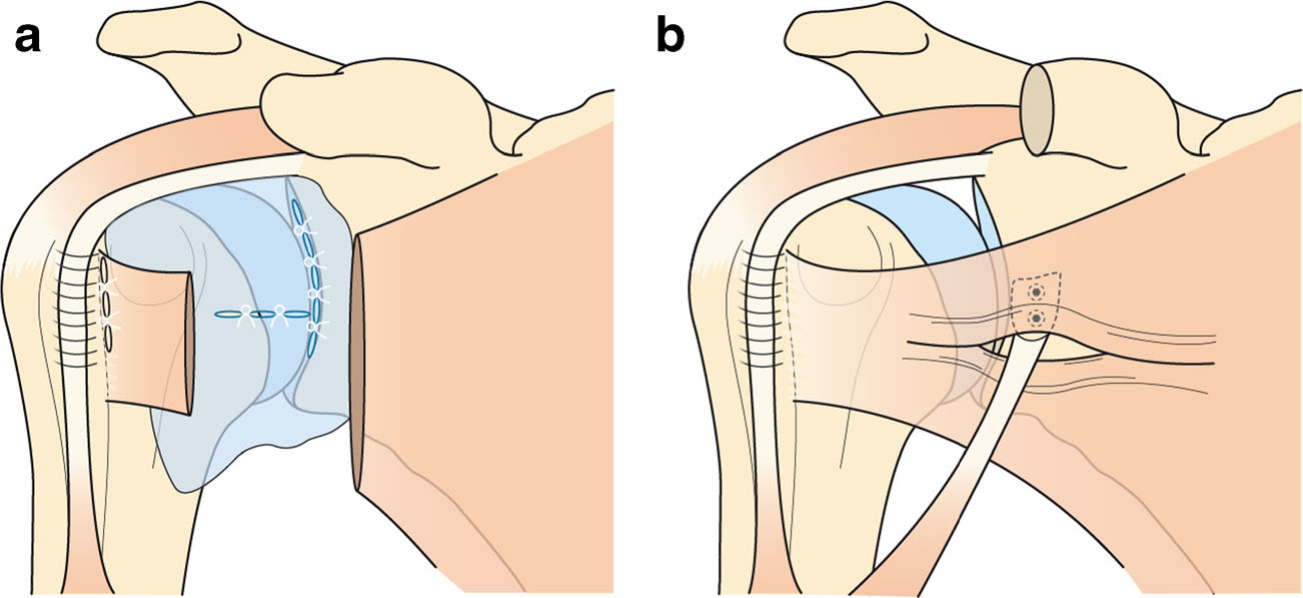
Schematic representations of surgical treatment of shoulder anterior instability: Bankart (**a**) and Latarjet (**b**) procedures

SSC tendon lesions have been described as a contributing factor to anterior glenohumeral dislocation, and vice versa. When associated with anterior instability, the middle and lower part of SSC tendon are injured [39]. Therefore, when investigating anterior instability, SSC tendon lesions must be investigated, because their repair is part of the treatment of recurrent anterior shoulder dislocations. Repair of the SSC tendon is therefore associated in the Bankart arthroscopic technique with repair of capsular, labral, and ligamentous lesions (Fig. 14a). Another surgical technique for anterior instability treatment, especially when there is associated glenoid bone loss, is the Latarjet procedure (Fig. 14b) (open surgery or arthroscopy) [40]. This procedure involves the removal and transfer of a section of the coracoid process with its attached muscle to the front of the glenoid. The Latarjet mechanism of stability is twofold:

- The coracoid acts as a bone block and increases the joint surface contact area.
- The conjoint tendon centralizes the humeral head into the joint.

### Coracoid (antero-medial) impingement

**Fig. 15:**
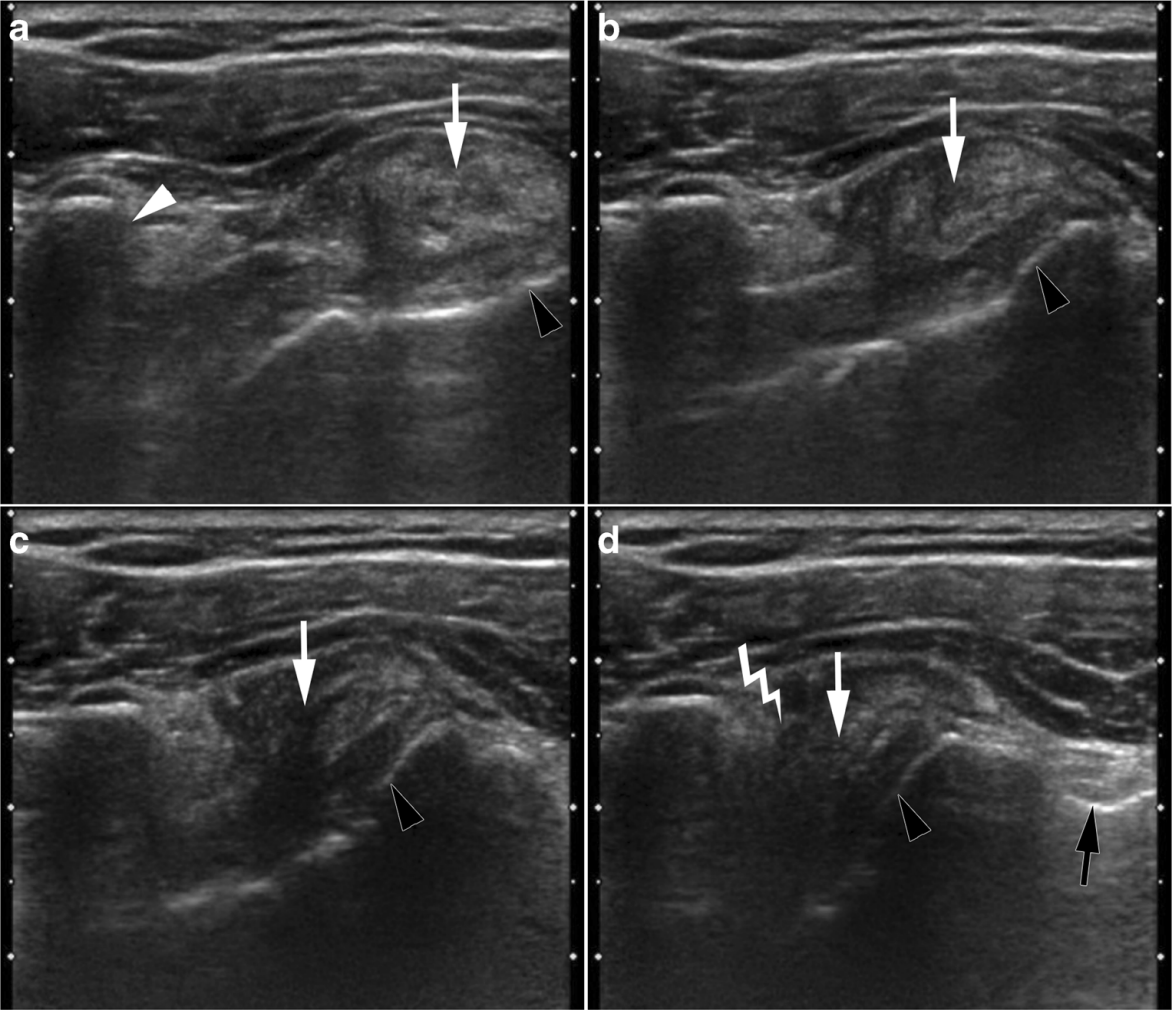
Axial dynamic ultrasound images during progressive internal rotation of the shoulder (from **a** to **d**). Anatomic landmarks are the coracoid process (white arrow head), the lesser tuberosity (black arrow heads) and the intertubercular sulcus (black arrow). A macrocalcification is seen in the SSC tendon (arrow). It gradually approaches the coracoid process (**b** and **c**). In internal rotation (**d**), pain and sensation of projection under the probe when calcification passes under the coracoid process, are consistent with coracoid impingement (lightning) associated with calcific tendinitis
  An accessory coraco-brachialis muscle is also a constitutional factor of CI, rarely visualized on ultrasound or MRI in front of the SSC [15].

Coracoid impingement (CI) results from impingement of the SSC tendon or LHBT between the coracoid process and the lesser tuberosity [41]. CI is quite uncommon, described in 5.1 % patients who underwent rotator cuff surgery in Suenaga’s study [42], but more frequent (19 %) in cases of extended rotator cuff tear (SSP, SSC and infraspinatus) [43] because of an anterior subdislocation of the humeral head (Fig. 13). Other contributing factors that have been described are:

- acquired factors: calcifications or ossification of the SSC tendon, mucoid cyst, tumour of the coracoid process, soft tissue tumour in the subcoracoid space, post traumatic (hypertrophic callus bone after proximal humerus, or coracoid process, or glenoid fractures) [44–47];
- iatrogenic factors: surgery of anterior or posterior instability, acromionectomy;
- constitutional factors: narrowness of the subcoracoid space. There is wide anatomical variability in the size and shape of the coracoid process. A coraco-glenoid space in the shape of a “round bracket” is associated with a short coraco-humeral distance and could therefore be a predisposing factor to CI syndrome [48]. The coraco-humeral distance (CHD) is defined as the shortest distance between the gleno-humeral head and the coracoid process on axial CT or MRI. A CHD <11 mm is sensitive but not specific for CI syndrome [49]. Dynamic ultrasound study is interesting, because the CHD decreases in internal rotation and elevation. The values of CHD associated with CI on ultrasound vary from 6 to 8 mm, according to authors [50, 51].
- A direct ultrasound sign of CI is the deformation of the bursal side of the SSC tendon when passing below the coracoid process, which can be associated with a sensation of projection under the probe (Fig. 15). Subcoracoid bursitis is an indirect sign of CI.

### Muscle injury

**Fig. 16:**
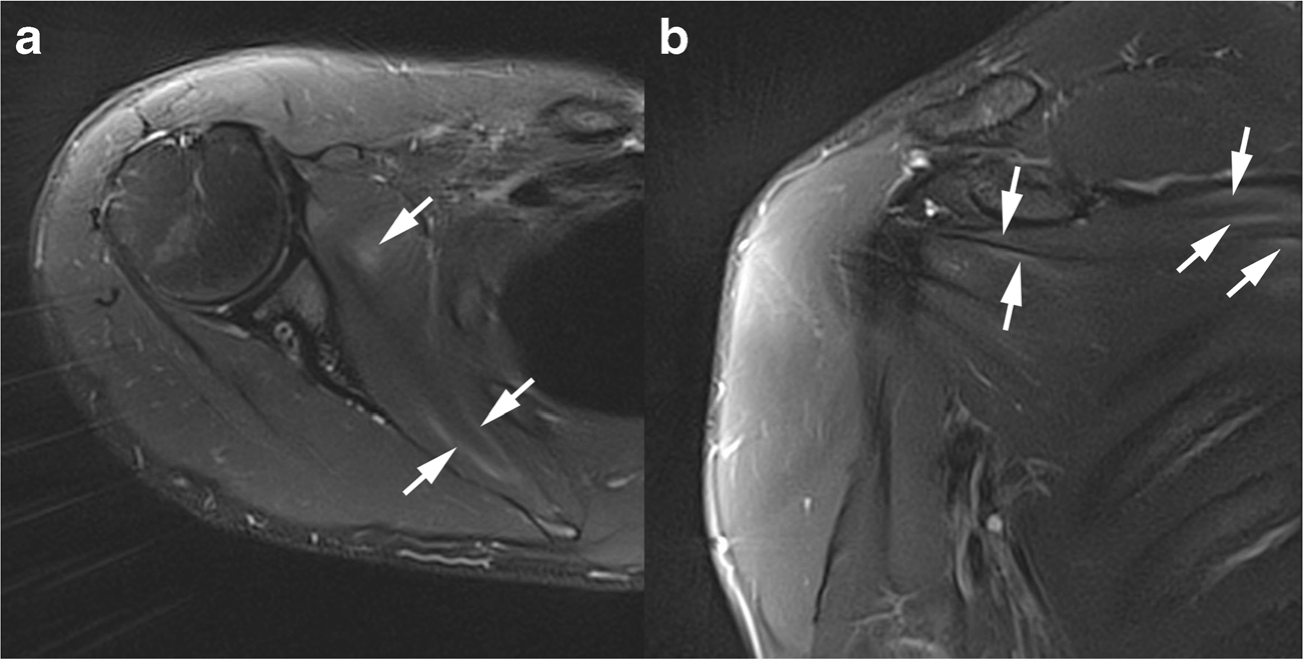
Right shoulder MRI of a 21-year-old military serviceman with sharp pain of the right shoulder, occurring after military training. Axial (**a**) and coronal oblique (**b**) FS DP weighted images. Mottled and linear high signal areas in the SSC muscle mostly at the myotendinous junctions (white arrows) are secondary to intrinsic muscle lesions

Throwing-related intrinsic lesions of the SSC muscle (Fig. 16) have been described, affecting mostly the inferior half of the SSC muscle at the myotendinous junction. These injuries occur mostly in baseball players having lower levels of dominant arm external rotation, leading to greater stretching of the SSC muscle [52].

## Conclusion

Although not well known and less common that SSP tendon lesions, SSC tendon lesions are more common than generally expected. The coupling of radiography with ultrasound yields an accurate diagnosis in most cases. Cross-sectional imaging techniques, especially CTA and MRA, allow accurate characterization of SSC tendon lesions, and identify their muscular impact. These elements are fundamental for the surgeon to adapt the surgical technique. Classically, SSC tendon tears are partial thickness tears starting superomedially and progressing inferolaterally. In most cases, SSC tendon tears are not isolated. Particular mechanisms, such as anterior instability or coracoid impingement, lead to specific injuries of the SSC tendon and specific surgical treatments. Intrinsic muscle injuries at the myotendinous junction have also been described.
